# Effects of Anti-TNF*α* Treatment on Mucosal Expression of IL-17A, IL-21, and IL-22 and Cytokine-Producing T Cell Subsets in Crohn's Disease

**DOI:** 10.1155/2018/3279607

**Published:** 2018-04-26

**Authors:** Anders Dige, Maria K. Magnusson, Claus Uhrenholt, Tue Kruse Rasmussen, Tue Kragstrup, Lena Öhman, Jens Dahlerup, Jørgen Agnholt

**Affiliations:** ^1^Gastro-Immuno Research Laboratory (GIRL), Department of Hepatology and Gastroenterology, Aarhus University Hospital, Aarhus, Denmark; ^2^Department of Microbiology and Immunology, Institute for Biomedicine, Sahlgrenska Academy, University of Gothenburg, Gothenburg, Sweden; ^3^Department of Biomedicine, Aarhus University Hospital, 8000 Aarhus C, Denmark; ^4^Department of Rheumatology, Aarhus University Hospital, 8000 Aarhus C, Denmark; ^5^Department of Internal Medicine and Clinical Nutrition, Institute for Medicine, Sahlgrenska Academy, University of Gothenburg, Gothenburg, Sweden

## Abstract

T helper 17 (Th17) cells produce interleukin (IL) 17-A. In addition, Th17 cells produce IL-21 and IL-22. Th17 cells have a disease-promoting role in Crohn's disease (CD). We investigated the effects of anti-TNF*α* treatment on mucosal gene expression (qPCR) of IL-17A, IL-21, and IL-22 as well as on the frequency of lamina propria (LP) T cell subsets producing these cytokines (flow cytometry) in 12 active CD patients before and after 4 weeks of anti-TNF*α* treatment with adalimumab. At baseline, in inflamed mucosa we found increased gene expression of IL-17A and IL-22 but not IL-21 when compared to noninflamed mucosa. There were increased frequencies of IL-21-producing LP T cells but no differences in the frequencies of IL-17A- or IL-22-producing LP T cells when comparing inflamed versus noninflamed mucosa at baseline. There were no changes in the mucosal gene expression of IL-17A, IL-21, and IL-22 or the frequencies of IL-17A-, IL-21- and IL-22-producing LP T cell subsets between baseline and following 4 weeks of adalimumab initiation. Our results do not support the hypothesis that anti-TNF*α* treatment has an early effect on the mucosal levels of IL-17A, IL-21, and IL-22 or LP T cell production of these cytokines in CD.

## 1. Introduction

Crohn's disease (CD) progresses due to a dysregulated mucosal immunological response towards the intestinal microflora in genetically susceptible individuals [1–3]. Interleukin (IL) 17-A-producing T helper (Th17) cells have been reported to play an important disease-promoting role in the progression of CD [4–7] because of their production of proinflammatory cytokines, which besides the hallmark cytokine IL-17A includes IL-21 and IL-22 [8]. However, these cytokines also have protective and regenerative effects on epithelial cells [9–11]. Consequently, the Th17 cells may have contradictory roles in CD, which may explain the inefficiency of anti-IL-17A antibodies as a treatment of CD [12]. Increased frequencies of IL-17-producing T helper cells and higher IL-17 mRNA expression have been observed at the mucosal level in CD patients compared to patients with infectious colitis [13] as well as healthy controls [6, 13–17]. A recent study reported that increased numbers of Th17 cells were associated with endoscopic disease activity in both CD and ulcerative colitis patients, and the Th17 cells were skewed towards concomitant production of interferon-*γ* [15]. The production of IL-21 and IL-22 is not specific to Th17 cells and has also been attributed to other CD4 T cell subsets, such as follicular T helper cells [18] and Th22 cells [19], respectively. Increased mucosal IL-21 expression has been observed in patients with active CD compared to ulcerative colitis patients and healthy controls. Increased numbers of IL-21- and IL-22-producing lamina propria (LP) T cells has also been reported in CD patients compared to healthy controls [16, 20].

Treatment with antibodies that neutralize the essential inflammatory cytokine tumor necrosis factor alpha (anti-TNF*α*) has become a mainstay in the treatment of CD [21]. However, the mechanisms of anti-TNF*α* efficacy are only partly elucidated. It has been proposed that the induction of apoptosis in LP T cells is important for anti-TNF*α* efficacy in CD treatment [22–24].

We previously reported that 26 weeks of anti-TNF*α* treatment was associated with a rise in the frequencies of circulating IL-17A- and IL-21-producing T cells [25]. Two studies from China reported that 10 weeks of anti-TNF*α* treatment was associated with a decreased mucosal gene expression of IL-17A and IL-21 and reduced frequencies of IL-17A- and IL-21-producing LP cells [26, 27]. However, because the clinical effect of anti-TNF*α* treatment often occurs one to two weeks following treatment initiation, it is difficult to decipher whether these observations are a bystander phenomenon to a general downregulation of the inflammatory response or a direct treatment mechanism.

We hypothesized that anti-TNF*α* treatment has an early (i.e., within 4 weeks of treatment initiation) effect on the mucosal IL-17A, IL-21, and IL-22 gene expression and the frequencies of mucosal IL-17A-, IL-21-, and IL-22-producing T cells in active CD. We aimed to test this hypothesis by measuring the mucosal gene expression of IL-17A, IL-21, and IL-22 as well as the cellular protein production of these cytokines in LP T cell subsets before and after 4 weeks of induction treatment with adalimumab. To clarify whether the cytokine levels were specific for the presence of active CD inflammation, we also included observations from areas of noninflamed tissue in the present study.

## 2. Methods

### 2.1. Patients and Samples

Twelve patients with active CD were included in this study. The patients had been diagnosed according to clinical, endoscopic, histopathological, and biochemical criteria [28]. Baseline patient characteristics are shown in Table 1. At inclusion, all patients exhibited clinical disease activity, as estimated by a Crohn's Disease Activity Index (CDAI) [29] greater than 150 or a Harvey-Bradshaw index (HBI) [30] of 4 or more. Furthermore, all included patients had biochemical signs of inflammation, for example, either elevated C-reactive protein (CRP) or increased fecal calprotectin levels. All patients displayed endoscopic disease activity at the inclusion endoscopy, which was evaluated using the Simple Endoscopic Score for Crohn's Disease (SES-CD) [31]. No patients were treated with anti-TNF*α* or corticosteroids or changed immunosuppressant dosing (azathioprine or methotrexate) within the 12 weeks prior to inclusion. Patients received standard induction dosing with subcutaneously administered adalimumab (AbbVie, North Chicago, Illinois), consisting of 160 mg at day 0, 80 mg at 2 weeks, and 40 mg at 4 weeks. All patients underwent a colonoscopy at day 0 and again one week postadministration of the fourth week of adalimumab dose. During the first colonoscopy, pinch biopsies from inflamed and noninflamed areas were obtained, and biopsies were taken from the same anatomical segments during the second colonoscopy. Blood samples were also drawn on the day of each colonoscopy.

### 2.2. Biochemical Parameters

Biochemical parameters (CRP and fecal calprotectin) were monitored at the times of blood sampling. All blood and fecal samples were analyzed by The Department of Clinical Biochemistry, Aarhus University Hospital, Aarhus, Denmark.

### 2.3. Analyses of Mucosal IL-17A, IL-21, and IL-22 Gene Expression

RNA was automatically isolated from paraffin-embedded mucosal biopsies using a QIAsymphony according to the manufacturer's protocol. The RNA concentrations and purity were determined using a NanoDrop 2000® 200 NanoQuant (Thermo Scientific). Predesigned primer and probe sets for IL-17A, IL-21, and IL-22 (Life Technologies, Darmstadt, Germany cat. number Hs00174383_m1, Hs00222327_m1, and Hs01574154_m1, resp.) labelled with the FAM-BHQ system as a fluorescence/quencher were used. RT-qPCR was performed on a 96-well StepOnePlus™ Real-Time PCR System (Life Technologies) using a 1-step protocol with TaqMan Gene Expression Assays. Samples were duplicated and the mean cycle threshold (C_T_) value was used for statistical analyses. Gene expression was standardized using the housekeeping gene HPRT-1, and data was analyzed using the delta-delta-Ct method as previously described [32].

### 2.4. Isolation of Lamina Propria Mononuclear Cells (LPMCs)

Biopsies were collected in ice-cold PBS and immediately placed on ice. Epithelial cells were removed by incubating the tissue for 15 minutes at 37°C with HBSS-EDTA (CMF HBSS supplemented with 2% AB serum, 1.5 mM Hepes (Gibco Life Technologies, Auckland, New Zealand)) and 2 mM EDTA (Thermo Fischer Scientific/Ambion, Waltham, Massachusetts) at three separate times, followed by a wash in RPMI 1640 that was supplemented with 10% AB serum and 1.5 mM Hepes. LPMCs were prepared via a 45- to 90-minute long incubation at 37°C with 125 *μ*l of collagenase (8 mg/ml) (Sigma-Aldrich, St Louis, Missouri) and 50 U/ml DNase I (Sigma-Aldrich), which were diluted in 5 ml RPMI 1640 supplemented with 10% AB serum and 1.5 mM Hepes. Following digestion, LPMCs were collected by filtration through a 70 *μ*m nylon mesh (BD Biosciences, San Jose, California) and analyzed using flow cytometry.

### 2.5. Flow Cytometry Staining and Analysis

The freshly isolated LPMCs were adjusted to a final concentration of 2 × 10^6^ LPMCs/ml in culture medium (RPMI 1640 with 10% pooled heat-inactivated human AB serum, 100 U/ml penicillin and 100 *μ*g/ml streptomycin) and incubated overnight at 37°C in a 5% CO_2_ atmosphere. The following day, the cells were stimulated with 0.1 *μ*g/ml ionomycin (Sigma-Aldrich, Denmark, cat. number I0634) and 5 *μ*g/ml phorbol 12-myristate 13-acetate (PMA) (Sigma-Aldrich, Denmark, cat. number P1585) in the presence of 10 *μ*g/ml brefeldin A (Sigma-Aldrich, Denmark, cat. number B7651) for 4 hours at 37°C in a 5% CO_2_ atmosphere. Then cells were harvested and 0.5 × 10^6^ cells in 100 *μ*l wash buffer (PBS, 2% bovine serum albumin (BSA) and 0.9% azide) were surface-stained with optimized amounts of antibodies against CD4 (anti-CD4-PerCP, BD Biosciences, cat. number 345770) and CD3 (anti-CD3-FITC, Biosciences, cat. number 555492) and Live/Dead Fixable Near-IR Dead cell stain kit (Life Technologies, cat. number L10119) according to the manufacturer's protocol. The surface staining was fixed with 1.5 ml BD FACS Lysing Solution (BD Biosciences, cat. number 349202). The cells were then permeabilized with 0.5 ml FACS Permeabilizing Solution 2 (BD Biosciences, cat. number 340973) and blocked with heat-inactivated mouse serum (Invitrogen, cat. number 10410) before staining with anti-IL-17A Alexa-647 (eBiosciences, cat. number 51-7179-42) and anti-IL-21 PE (eBiosciences, cat. number 12-7219-42) or anti-IL-22 PE (R & D, cat. number IC7821P). Finally, the cells were fixed in 250 *μ*l PBS with 1% formaldehyde. Five-color flow cytometry was performed within 24 hours and 10^5^ events in the forward-side scatter lymphocyte gate were recorded. The combination of forward-scatter-height and forward-scatter-area was used to exclude the events without single cell appearances. Live/dead stain was used to exclude nonviable cells from analysis. The stimulation of LPMCs was associated with a distinct downregulation of CD4, thereby prohibiting the possible identification of CD4^+^ CD3 cells. Instead, we only gated the CD3^+^ events for the analyses of intracellular IL-17A, IL-21, and IL-22 production. The gating for IL-17A, IL-21, and IL-22 was based on combined isotype and fluorescence-minus-one controls (Supplementary Figure 1). Flow cytometry was performed using a FACSCanto flow cytometer (BD Biosciences), and data was analyzed using FACS Diva 5.1 software (BD Biosciences). The staining procedure failed in four of the included patients at baseline and in three patients at the 4-week follow-up appointment. Paired samples (baseline/week 4) were therefore only available in seven of the included patients, who all responded to anti-TNF*α* treatment.

### 2.6. Statistical Analyses

Data is presented as medians with interquartile ranges (IQR). A Wilcoxon signed-rank test was used to evaluate the differences between two sets of paired samples using GraphPad Prism 6.0 (GraphPad Software, La Jolla, USA). *p* values < 0.05 were considered statistically significant.

### 2.7. Ethical Considerations

This study conformed to the Declaration of Helsinki. All participants provided a written, informed consent. The study protocol was approved by the Central Denmark Region Committee on Biomedical Research Ethics (journal number M-20100216).

## 3. Results

### 3.1. Clinical Effects of Adalimumab Treatment

Adalimumab treatment improved endoscopic disease activity scores. The SES-CD scores decreased from 15 (9–16) at baseline to 5 (2–8) at week 4 (*p* = 0.003). Two patients exhibited normal mucosa (SES-CD value = 0) at week 4. The disease activity scores reduced from a CDAI level of 279 (235–298) at baseline to 102 (61–133) at week 4 (*p* = 0.002). The level of HBI decreased from 10 (8–11) at baseline to 3 (1–5) at week 4 (*p* = 0.002). Furthermore, CRP levels decreased from 4.8 mg/L (1.2–11) at baseline to 0.9 mg/L (0.6–4.1) at week 4 (*p* = 0.01). Fecal calprotectin levels decreased from 495 mg/kg at baseline to 138 mg/kg (30–770) at week 4 (*p* = 0.10). One of the included patients (patient number 7) did not respond to adalimumab treatment and experienced an increased SES-CD score (1 point) at week 4. This patient experienced only a slight reduction in disease activity from baseline to week 4 (CDAI-score: 232–213; HBI-score: 12–10).

### 3.2. Increased Gene Expression of IL-17A and IL-22 and Increased Frequency of IL-21-Producing T Cells in Inflamed CD Mucosa

**Figure 1:**
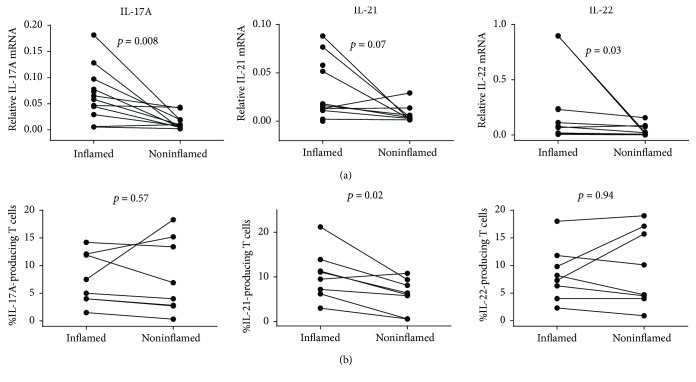
Mucosal gene expression and LP T cell producing IL-17A, IL-21, and IL-22 at baseline in inflamed and noninflamed mucosa. Gene expression was determined by rtPCR (a) and frequencies of IL-17A-, IL-21-, and IL-22-producing cells among LP CD3^+^ T cells were determined by flow cytometry (b). Gene expression data is displayed as the normalized ratios between the relative expression of the gene of interest and the housekeeping gene *HPRT-1*. Wilcoxon signed-rank test for comparison was applied on paired samples (rtPCR: IL-17A *n* = 11; IL-21 *n* = 9; IL-22 *n* = 9; flow cytometry: *n* = 8 for each cytokine).

All samples from inflamed mucosal areas had detectable IL-17A, IL-21, and IL-22 gene expression. However, this was not true for all noninflamed tissue samples. Therefore, we censored below the detection IL-17A expression data from 1 patient and IL-21 and IL-22 expression data from 3 patients from the comparison graphs and statistical analyses presented in Figure 1(a). Baseline gene expressions of IL-17A and IL-22 were higher in mucosal areas with active inflammation compared to noninflamed mucosal areas in the same individual (*p* = 0.008 and *p* = 0.03, resp., Figure 1(a)). However, the mucosal gene expression of IL-21 only tended to be higher (*p* = 0.07) in inflamed mucosal areas compared to noninflamed mucosal areas (Figure 1(a)).

Flow cytometry analyses were performed in eight patients at baseline. The frequencies of IL-17A- and IL-22-producing T cells among LP cells were 6.3% (4–12) and 7.8% [5.2–10.8], respectively, in areas with active inflammation. These frequencies did not differ from the baseline levels in noninflamed areas of the intestine {(IL-17A 5.5% [2.8–14%], *p* = 0.57), (IL-22 (7.4% [4.3–16%] *p* = 0.94)}. There was an increased frequency of IL-21-producing LP T cells in inflamed areas of the intestines versus the frequencies of these cells in noninflamed areas of the intestines at baseline (10.3% [6.7–13%] versus 6.2% [3.2.–8.8%], *p* = 0.02) (Figure 1(b)). Flow cytometry analyses revealed that the production of IL-17A, IL-21, and IL-22 among LPMC was only present in CD3-expressing cells within the applied lymphocyte gate. There were no differences in the frequencies of LP CD3^+^ T cells between areas with active inflammation (54% [48–59%]) compared to noninflamed area (51% [49–53%]) (*p* = 0.33).

### 3.3. Four Weeks of Adalimumab Treatment Does Not Change Mucosal Gene Expression or LP T Cell Production of IL-17A, IL-21, and IL-22

**Figure 2:**
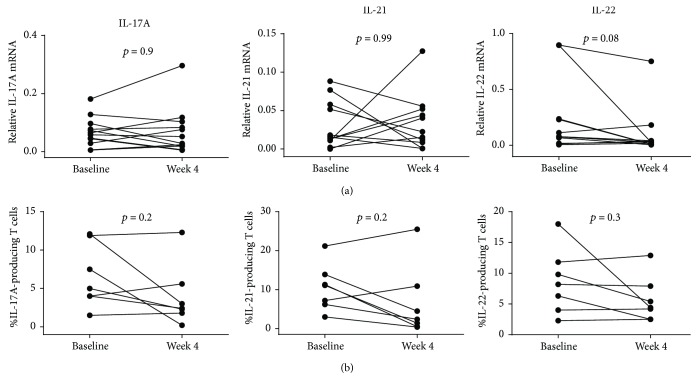
Comparison of mucosal gene expression and LP T cells expressing IL-17A, IL-21, and IL-22 between baseline and week 4 of adalimumab treatment. Gene expression was determined by rtPCR (a) and frequencies of IL-17A-, IL-21-, and IL-22-producing cells among LP CD3^+^ T cells were determined by flow cytometry (b). Gene expression data is displayed as the normalized ratios between the relative expression of the gene of interest and the housekeeping gene *HPRT-1*. Wilcoxon signed-rank test for comparison was applied on paired samples (rtPCR: IL-17A *n* = 12; IL-21 *n* = 11; IL-22 *n* = 11; flow cytometry: *n* = 7 for each cytokine).

After 4 weeks of adalimumab treatment, new biopsies were obtained from the same areas of the intestine that were inflamed at inclusion. Gene expression of IL-17A was detectable in all obtained samples, whereas the IL-21 and IL-22 expression levels were below the detection limit in one sample, which was censored from subsequent analyses. There were no changes in mucosal gene expression of IL-17A (*p* = 0.88) or IL-21 (*p* = 1.0) compared to gene expression in the biopsies obtained from inflamed areas at baseline. Following adalimumab treatment, the IL-22 gene expression (*p* = 0.08) trended lower compared to baseline (Figure 2(a)).

Moreover, there were no changes in the frequencies of IL-17A-, IL-21-, or IL-22-producing LP T cells after 4 weeks of adalimumab treatment [3.0% (2.3–5.8%) IL-17A (*p* = 0.20), 4.5% (1.4–10.9%) IL-21 (*p* = 0.18), and 5.4% (4.2–7.9%) IL-22 (*p* = 0.24)] compared to the inflamed area at baseline (Figure 2(b)) (paired flow cytometry data were only available from seven patients). The same comparison revealed no change in the frequency of LP CD3^+^ T cells at week 4 of adalimumab treatment [52% [46–57%] (*p* = 0.61)]. Post hoc censoring of the single patient (patient number 7) who did not respond to the adalimumab treatment did not affect the statistical interpretations of the results.

## 4. Discussion

Our study investigated the effects of anti-TNF*α* treatment on mucosal gene expression of IL-17A, IL-21, and IL-22 and LP T cell production of these cytokines in active CD. The data showed that clinical response to anti-TNF*α* treatment did not change these parameters. However, when comparing mucosal areas with active inflammation to noninflamed mucosal areas at baseline, we did observe increased gene expression of IL-17A and IL-22 as well as increased frequencies of IL-21-producing LP T cells.

The presence of mucosal Th17 cells and the gene expression of IL-17A are associated with CD inflammation. In general, Th17 cells have been considered as disease promoting in the progression of CD [4–6, 33]. Several studies have substantiated this by reporting increased Th17 cell levels and IL-17A gene expression levels in CD patients compared to healthy controls [6, 13–17]. A recent study also reported increased levels of mucosal Th17 cells to be associated with the endoscopic disease activity [15]. However, Hueber et al. found that anti-IL-17A treatment was associated with disease deterioration in some patients, suggesting that Th17 cells may also have an anti-inflammatory role in CD [12]. In agreement with findings by others, we observed increased IL-17A and IL-22 gene expression in inflamed CD mucosa compared to noninflamed mucosa at baseline [6, 9, 13, 14, 17]. However, this was not reflected at the cellular level as there were no differences in the frequencies of IL-17A- or IL-22-producing LP T cells between inflamed and noninflamed areas. This observation indicates that even though the same frequency of T cells expressing IL-17A and IL-22 are present in inflamed and noninflamed mucosa, the relative gene expression is higher during inflammation. This could reflect cytokine production in non-T cells such as innate lymphoid cell type 3 (ILC3), although our analyses indicated that the cytokine expression was limited to CD3 expressing lymphocytes. Globig et al. [15] and Jiang et al. [16] each demonstrated an increase in IL-17A-producing LP T cells in active CD versus mild/quiescent CD versus healthy controls. However, these studies did not include an individual comparison between inflamed and noninflamed CD as performed here. Furthermore, neither of these studies measured longitudinal changes as reported here. In contrast to Globig et al. and Jiang et al., yet similar to our findings, Leung et al. did not observe a difference in the frequency of IL-17-producing cells when comparing individual levels of inflamed and noninflamed CD mucosa [34]. Additionally, we did not detect any difference in the frequency of IL-22-producing LP T cells when comparing the levels of inflamed with noninflamed mucosa. One study demonstrated that the frequency of IL-22-producing LP T cells correlates with endoscopic disease activity in CD [16]; however, this study did not include an intraindividual comparison as performed here. In accordance with findings in other studies, we observed a higher level of IL-21-producing LP T cells in inflamed versus noninflamed mucosa at baseline [34, 35].

In contrast to our hypothesis, we did not observe any changes in the gene expression, in inflamed mucosa, of IL-17A, IL-21, or IL-22 as a result of anti-TNF*α* treatment. Neither did we observe any differences at the cellular level with respect to protein expression of these cytokines in LP CD3^+^ cells during anti-TNF*α* treatment despite a marked visual improvement upon endoscopic examination and reduced clinical disease activity during treatment. However, the conclusion regarding the cellular level is based on seven patients. We investigated the frequency and not the absolute cell number of cytokine-producing CD3^+^ LP T cells to detect any specific effects of anti-TNF*α* treatment on these cells. If we had studied the absolute numbers, however, we would expect to find a marked difference between inflamed and noninflamed tissue as the absolute numbers of inflammatory cells expands greatly with inflammation.

Our results are in contrast to the data reported by two studies demonstrating that 10 weeks of anti-TNF*α* treatment in Asian CD patients were associated with decreased gene expression of IL-17A and IL-21 [26, 27]. Furthermore, one of these studies reported decreased frequencies of IL-17A- and IL-21-producing LP cells from anti-TNF*α* treatment, without specifically limiting their analyses to the T cells [26]. This discrepancy can be related to genetic differences between Asian patients and the Caucasian patients included in the present study; however, a plausible explanation is the varying time points for follow-up examination in the studies (10 versus 4 weeks, resp.). We observed a marked endoscopic improvement following 4 weeks of treatment, which supports that the anti-inflammatory effects of this treatment are well established at this time point. Thus, a 10-week interval makes it intrinsically difficulty to decipher between ameliorated inflammation and the effects of anti-TNF*α* when examining mucosal IL17-A and IL-21 production. Consistent with needing to perform examinations earlier in the treatment course, we recently reported changes in innate immune responses from 4 weeks of anti-TNF*α* treatment using this same study cohort and investigational time points [36]. In that analysis, we observed reduced numbers of mucosal macrophages with intermediate HLA-DR expression and increased numbers of CD103^+^ dendritic cells [36]. Our previous and current results together suggest that anti-TNF*α* treatment has a more prominent effect on innate immunity at a short interval as compared to adaptive immunity.

In conclusion, if modulation of IL-17A, IL-21, and IL-22 were mechanistically responsible for the clinical efficacy of anti-TNF*α* treatment, our analyses should have revealed differences between inflamed mucosa before and after 4 weeks of anti-TNF*α*. Since this was not the case, our data do not support this hypothesis.

## Figures and Tables

**Table 1:** Baseline characteristics.

Patient number	Gender	Immunosuppressant	Behavior	Location	Smoking status	CDAI	HBI	SES-CD	CRP	Fecal calprotectin
1	M	Yes	Stricturing	Ileal	No	301	6	8	8	576
2	F	Yes	Penetrating	Colonic	No	266	10	8	0.6	148
3	M	No	Nonstricturing, nonpenetrating	Colonic	No	153	4	16	3.2	1215
4	F	No	Nonstricturing, nonpenetrating	Colonic	No	299	8	11	51.7	>3600
5	M	Yes	Stricturing	Ileocolonic	No	292	10	17	0.8	405
6	M	Yes	Nonstricturing, nonpenetrating	Ileocolonic	Yes	315	10	7	9.8	941
7	M	No	Nonstricturing, nonpenetrating	Colonic	No	232	12	15	1.4	Missing
8	F	Yes	Nonstricturing, nonpenetrating	Colonic	No	179	7	18	3.8	342
9	F	Yes	Nonstricturing, nonpenetrating	Colonic	No	251	12	14	12.0	495
10	F	Yes	Nonstricturing, nonpenetrating	Colonic	No	296	10	10	5.7	178
11	M	Yes	Nonstricturing, nonpenetrating	Colonic	No	238	11	15	0.9	211
12	M	No	Stricturing	Ileocolonic	No	295	9	15	36.5	>3600

CRP: C-reactive protein in mg/L, reference < 8 mg/L; Fecal calprotectin, reference < 50 mg/kg.

## Supplementary Materials

Supplementary Materials Figure 1: gating strategy for identification of cytokine-producing LP T cells. Isolated LP cells were identified by their forward- and side-scatter appearance (a). Events without single cell appearances were excluded by the combination of forward-scatter-height and forward-scatter-area. Live/dead stain was used to exclude nonviable cells from analysis (b). T cells were identified by their expression of CD3 (c). The lymphocyte gate for cytokine-production was set by a combination of isotype- and fluorescent-minus-one controls (d). This gate was then applied on LP cells stained for cytokine expression (in this example IL-17A and IL-22) (e).
